# The chromosomal translocation *t*(1;6)(p35.3;p25.2), recurrent in chronic lymphocytic leukaemia, leads to *RCC1::IRF4* fusion

**DOI:** 10.1111/bjh.19790

**Published:** 2024-10-15

**Authors:** Sandrine Jayne, Cristina López, Natalie Put, Inga Nagel, Els Lierman, Eva Maria Murga Penas, Lucienne Michaux, Matthew J. Ahearne, Harriet S. Walter, Susanne Bens, Cosima Drewes, Monika Szczepanowski, Matthias Schlesner, Philip Rosenstiel, Iwona Wlodarska, Reiner Siebert, Martin J. S. Dyer

**Affiliations:** ^1^ The Ernest and Helen Scott Haematological Research Institute, Leicester Cancer Research Centre University of Leicester Leicester UK; ^2^ Institute of Human Genetics, Christian‐Albrechts‐University Kiel and University Hospital Schleswig‐Holstein—Campus Kiel Kiel Germany; ^3^ Institute of Human Genetics Ulm University and Ulm University Medical Center Ulm Germany; ^4^ Institut D'Investigacions Biomèdiques August Pi I Sunyer (IDIBAPS) Barcelona Spain; ^5^ Centro de Investigación Biomédica en Red de Cáncer (CIBERONC) Madrid Spain; ^6^ Universitat de Barcelona Barcelona Spain; ^7^ Center for Human Genetics University Hospitals Leuven Leuven Belgium; ^8^ Department of Oncology, Hematology and Radiotherapy Ziekenhuis Oost‐Limburg Genk Belgium; ^9^ Limburgs Oncologisch Centrum Genk Belgium; ^10^ Faculty of Medicine and Life Sciences UHasselt—Hasselt University Diepenbeek Belgium; ^11^ Hematopathology Section Christian‐Albrechts‐University Kiel Kiel Germany; ^12^ Second Medicine Department University Hospital Schleswig‐Holstein, Campus Kiel Kiel Germany; ^13^ Bioinformatics and Omics Data Analytics (B240) German Cancer Research Center (DKFZ) Heidelberg Germany; ^14^ Biomedical Informatics Data Mining and Data Analytics, University of Augsburg Augsburg Germany; ^15^ Institute of Clinical Molecular Biology Christian‐Albrechts‐University Kiel Germany

**Keywords:** chromosomal translocation, CLL, IRF4

## Abstract

The chromosomal translocation *t*(1;6)(p35.3;p25.2) is a rare but recurrent aberration in chronic lymphocytic leukaemia (CLL). We report molecular characterization of 10 cases and show that this translocation juxtaposes interferon regulatory factor 4 (*IRF4)* on 6p25 with regulator of chromosome condensation 1 (*RCC1)* on 1p35. The breakpoints fell within the 5′ untranslated regions of both genes, resulting in *RCC1::IRF4* fusion transcripts without alterations of the protein‐coding sequences. Levels of expression of both RCC1 and IRF4 proteins were not obviously deregulated. The cases showed other mutations typical of CLL and we confirm previously reported skewing towards the *IGHV*‐unmutated subtype. *RCC1::IRF4* fusion characterizes a rare subset of CLL.

## INTRODUCTION

Unlike other lymphoid malignancies, balanced, reciprocal chromosomal translocations are rare in chronic lymphocytic leukaemia (CLL).^1^ However, chromosomal translocation *t*(1;6)(p35.3;p25.2) has been previously described as a rare but recurrent event.^2^, ^3^, ^4^, ^5^ The presence of this translocation in 12 previously reported cases of CLL has been associated with high‐risk chromosomal aberrations like del(11q) and del(17p), unmutated *IGHV* status and a progressive clinical course (for review, see Table S1).

Here, we have characterized 10 further cases of CLL with *t*(1;6)(p35.3;p25.2) and show that this translocation involves directly and recurrently the gene‐encoding interferon regulatory factor 4 (*IRF4*) on chromosome 6p25.2 with the regulator of chromosome condensation 1 (*RCC1*) gene on chromosome 1p35.3. Moreover, we provide insights into the genome‐wide mutational landscape as well as the expression of the two involved proteins in CLL cases carrying this recurrent aberration.

## MATERIALS AND METHODS

This study was approved by local Research Ethics Committee and the University Hospitals of Leicester NHS Trust (06/Q2501/122), by the Institutional Review Board of the Medical Faculty of the University of Kiel (A150/10), University of Ulm (349/11) and University of Leuven (B322201111374). Samples were obtained after written informed consent.

Details of experimental methods are described in the Supporting Information.

## RESULTS AND DISCUSSION

We identified 10 previously unreported patients with CLL across our three centres in which karyotyping revealed a *t*(1;6)(p35.3;p25.2). In line with the 12 previously reported cases (Table S1), the *t*(1;6) appeared as the sole or communal cytogenetically detectable event in 9 of the 10 cases suggesting that it occurred early in disease pathogenesis. Karyotypes and clinical details are shown in Table 1 and Figure S1. The median age at diagnosis of the patients was 66.5 years. In line with previous report,^2^ there was a skewing (4/6 cases, 67%) towards an unmutated *IGHV* status although two of the new cases with available data had mutated *IGHV*. While some patients in this and previously reported cohorts experienced adverse clinical outcomes (Richter transformation or refractory disease), it is not clear due to the relatively small number of patients and co‐occurring adverse genetic aberrations (trisomy 12/*NOTCH1* mutations, del(11q)) whether *t*(1;6) translocation alone is associated with a particularly aggressive clinical course; however, seven patients with available clinical data (over 10‐year follow‐up) required therapy (data not shown).

**TABLE 1:** Clinico‐biological characteristics of the cohort and genomic data of the *t*(1;6) translocation.

Case	ICGC case	Age at diagnosis	Gender	Stage (Binet)	WCC (x10^9^/L)	TTFT	*IGHV*	*%IGHV mut*	Conventional and molecular cytogenetics						Genomic data (WGS or PCR) (hg19)								
									Karyotype	*RCC1::IRF4 fusion*	13q	*12*	*11q*	*17p*	SVs type	Chr1	Pos.1	Gene1	Site	Chr2.	Pos2.	Gene2	Site
1	4 154 480	42	F	C	60.5	16	1–69	100	46, XX, *t*(1;6)(p35;p25), add(3)(p26)[11]/47, idem, +12[9]	F	‐	gain	‐	‐	T	1	28 835 006	*RCC1*	Int 2	6	392 127	*IRF4*	Int 1
2	4 153 126	58	M	nk	nk	nk	4–34	100	46, XY, *t*(1;6)(p35;p25), del(13)(q13q14)[20]	F	del	‐	‐	‐	T	1	28 834 982	*RCC1*	Int 2	6	392 208	*IRF4*	Int 1
3	4 149 243	70	M	nk	24.4	1240	3–30	98–100	45, XY, *t*(1;6)(p35;p25), dic(14;17)(q10;q10)[2]/45, XY, idem, *t*(3;12)(p22;p13)[3]/45, XY, idem, del[10](q26), add(16)(q25)[cp3]/45, XY, idem, der(8)*t*(8;11)(q24;q13)[2]/46, XY[5]		del	‐	gain	del^a^	T	1	28 834 384	*RCC1*	Int 1	6	392 526	*IRF4*	Int 1
4							nk		*t*(1;6)(p35;p25)	F	del	‐	‐	‐	nd								
5		73	F	A	12	590	3–30	M	47, XX, +12[2]/47, idem, *t*(1;6)(p35;p25)[7]/46, XX[11]		‐	gain	‐	‐	T	1	28 507 477	*RCC1*	Int 1	6	393 084	*IRF4*	Int 1
6		63	M	C	11	nk	4–31	M	46, XY, *t*(1;6)(p35;p25)[10]		‐	‐	‐	del	T	1	28 508 915	*RCC1*	Int 1	6	393 001	*IRF4*	Int 1
7		70	M	B	45	418	1–69	100	46, XY, (1;6)(p35;p25)[10]		‐	‐	‐	‐	nd								
8		70	M	B	nk		nk		47, XY, *t*(1;6)(p35;p25),+15[6]/46, XY[11]		‐	‐	‐	‐	NA								
9		55	M	A	6.6		nk		46, XY, *t*(1;6)(p35;p25)[7]/46, XY[9]		‐	‐	‐	‐	NA								
10		74	M				nk		46, XY, *t*(1;6)(p34;p24)[3]/46, XY, *t*(1;6)(p34;p24), add(17)(p13)[3]/46, XY[4]		nk	nk	nk	nk	nd								
L1															T	1		*RCC1*	Int 3	6		*IRF4*	Int 1
L2															T	1		*RCC1*	Int 1	6		*IRF4*	Int 1
L3															T	1	28 508 442	*RCC1*	Int 2	6	392 172	*IRF4*	Int 1
L4															nd								

*Note*: Cases L1–L4 have been previously described in.^2^ *IRF4* break‐apart assays confirmed the breaks in Cases 1–4. *RCC1* break‐apart and *RCC1::IRF4* dual‐colour dual‐fusion assays were only performed in Cases 1, 2 and 4 and verified the *RCC::IRF4* fusion in these cases.

Abbreviations: Chr., chromosome; del, deletion; M, mutated, WCC, white cell count; NA, DNA not available; N, normal; nd, not detected by PCR; Nk, not known; Pos., position; SVs, structural variants; T, translocation; TTFT, time to first treatment in days, F, fusion.

^a^ *TP53* mutation for Case 3.

Interphase fluorescence *in situ* hybridisation (FISH) was performed using dual‐colour *IRF4* break‐apart probe to confirm *IRF4* as a translocation partner in 6p25 (Figure S1). The 1p35.3 breakpoint was mapped to a 500‐kb region between BACs RP11‐290H1 and RP11‐442 N24 and the 6p25.2 breakpoint into the BAC RP11‐233 K4. The 500‐kb region of 1p35 encompasses four genes including *RCC1* (Figure S2). *IRF4* break‐apart assays confirmed the breaks in Cases 1–4. *RCC1* break‐apart and *RCC1::IRF4* dual‐colour dual‐fusion assays were performed in Cases 1, 2 and 4 (Table 1) and verified the *RCC::IRF4* fusion in these cases.

**FIGURE 1:**
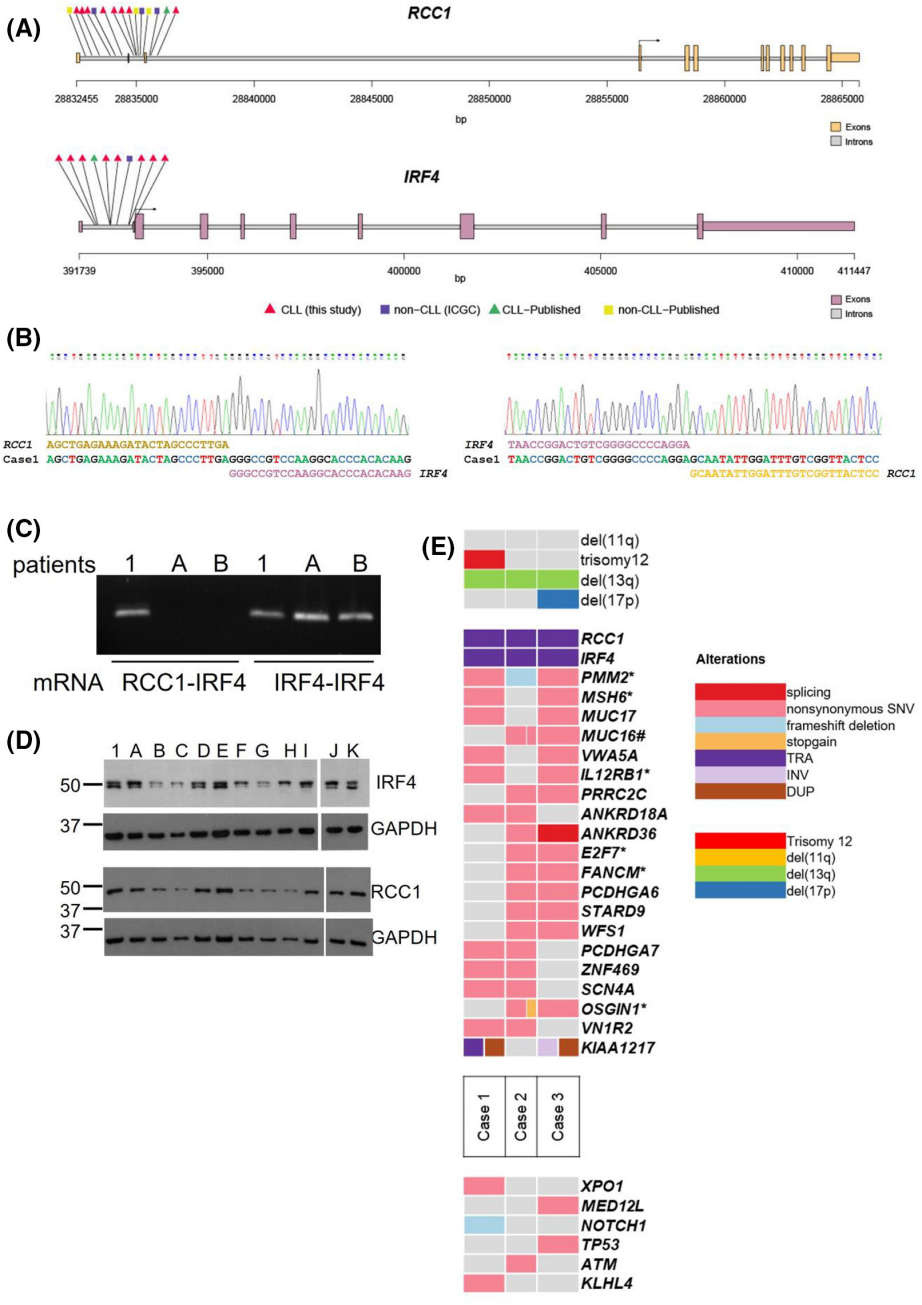
Identification of *RCC1* as a partner of *IRF4* in the *t*(1;6)(p35.3;p25.2) translocation. (A) Translocation breakpoints identified in *RCC1* and *IRF4* locus in CLL described in this study (red triangle), CLL previously published (green triangle),^19^ non‐CLL cases from ICGC (purple rectangle) and non‐CLL cases previously published (yellow rectangle).^18^, ^20^ (B) Sanger sequencing of the fusion breakpoint between *RCC1* and *IRF4* in Case 1. (C) RT‐PCR analysis using *RCC::IRF4* or *IRF4‐IRF4* primers pairs showed the expression of a chimaeric mRNA in Case 1 and not in patients A and B without the translocation. (D) Western blot analysis for the expression of IRF4 and RCC1 in patient 1 and 11 CLL patients without the translocation. (E) The upper part displays the genes recurrently affected by SNVs, SVs and indels in *RCC1::IRF4* patients. The events in recurrently affected genes are shown independent of the transcript form. The genes are ordered by frequency. The cases are also annotated with del(11q), trisomy 12, del(13q) and del(17p) chromosomal aberrations analysed by ACEseq. Moreover, in the part below, genes altered and previously described in CLL but not recurrent in the *RCC1::IRF4* patients. Annotated with * mutated genes that could be germline variants and with # altered genes located in late replicated regions.

Samples from three patients (Cases 1–3) were subjected to whole‐genome sequencing using the ICGC MMML‐Seq pipelines which confirmed the *RCC1::IRF4* juxtaposition in all. The genomic breakpoints in *RCC1* locus were located in intron 1 in Case 3 and intron 2 in Cases 1 and 2. The breakpoints within the *IRF4* locus were all located in intron 1 (Figure 1A, Table 1). Targeted Polymerase chain reaction (PCR) and subsequent sequencing confirmed the *RCC1::IRF4* genomic fusion on all three cases subjected to whole‐genome sequencing, as well as on Cases 5 and 6 (DNA was unavailable for Cases 8 and 9), and additionally on three cases (L1–L3) from previous report.^2^ Whereas the breakpoints were located within intron 1 in all tested cases of *IRF4* (Figure 1A,B, Table 1), the breakpoints in *RCC1* were variable involving intron 1 (three cases), intron 2 (three cases) and intron 3 (two cases). The sequences at the breakpoint junctions were different in all cases, with no obvious adjacent recombinogenic signal sequences. Targeted PCR failed to amplify the fusion in four cases (three new cases [Cases 4, 7 and 10] and Case L4 from previous report^2^) which could be due to complex aberrations, location of the breakpoints elsewhere in regions analysed by the FISH assays or genomic mutations affecting primer binding. Indeed, analysis of the genomic sequencing data of Cases 1–3 identified mutations affecting intron 3 and intron 1 of *RCC1* in Case 2 and Case 3 respectively. We also detected intronic mutations affecting introns 1 and 3 of the *IRF4* gene in Cases 2 and 3 respectively (Table S2). Nevertheless, in contrast to other lymphoma subtypes with *IRF4* rearrangements,^6^ coding mutations in *IRF4* were absent.

The 5’ untranslated region (UTR) of *RCC1* (which also hosts the long non‐coding RNA *SNHG3*) encompasses exon 1 to exon 3, whereas the 5’ UTR of *IRF4* only comprises exon 1. Therefore, the translocation does not generate a chimaeric RCC1::IRF4 protein but rather a swapping of regulatory regions mapping 5′ of the translation start and the generation of chimaeric mRNAs with swapped 5’ UTRs but conserved open reading frame (ORF). Reverse transcription polymerase chain reaction (RT‐PCR) analysis performed using *RCC1*‐5’‐UTR/*IRF4* 5′‐coding regions primers confirmed the presence of the chimaeric *RCC1::IRF4* mRNA in a patient presenting with the translocation (Case 1) but not in CLL samples without the translocation. Ongoing transcription of the non‐translocated *IRF4* allele in the patient with the translocation was also confirmed (Figure 1C). Expression of both proteins was at levels comparable between one patient with the translocation (Lane 1) and patients lacking the translocation (Lanes A–K) (Figure 1D). Extending this analysis to a panel of 35 CLL cases without translocation, both proteins exhibited variable expression (data not shown) but without any obvious relationship to either clinical or laboratory prognostic factors (data not shown).

The translocation was the sole cytogenetic aberration in 3/10 patients (Cases 7, 8 and 9), 2/10 had concomitant trisomy 12 (Cases 1 and 5), 2/10 del(13q) (Cases 2 and 4), 1/10 del(17p) (Case 6) and 1/10 both del(13q) and del(17p) (Case 3) (Table 1). Exploring the whole genome sequencing (WGS) data from Cases 1–3, trisomy 12 was confirmed in Case 1 and del(17p) in Case 3. Del(13q) was observed in all three cases but was not identified by FISH in Case 1. To investigate the genes involved in this subset of *RCC1::IRF4*‐positive CLL cases, we performed an integrative analysis of the different somatic genomic alterations, namely single nucleotide variant (SNVs), indels and structural variants (SVs) with a total of 22 coding genes and 25 non‐coding genes recurrently altered identified (≥2 cases). In our *RCC1::IRF4* CLL cohort, we did not identify recurrent mutations in the commonly mutated genes previously described in CLL.^7^, ^8^, ^9^ Nevertheless, some commonly mutated genes in CLL, like *TP53, NOTCH1, XPO1* and *ATM*, were also mutated in our cohort but affected only one patient (Figure 1E; Table S3). Other mutations included *PMM2*, *MSH6*, *E2F7*, *FANCM*, *OSGIN1* and *IL12RB1* (Figure 1E; Table S4) although these mutations could be germline as constitutional DNA was not available. Selected mutations were validated using PCR and Sanger sequencing (Table S5).

Next, we investigated the DNA methylation at 5 CpGs used to classify CLL subgroups in Cases 1–3. These three cases were classified as naive CLL according to their DNA methylation pattern, correlating with their unmutated *IGHV* status (Figure S3). Moreover, we interrogated the DNA methylation at the *RCC1* and *IRF4* loci across normal B‐cell differentiation and between CLL with or without the *RCC1::IRF4* translocation (Figure S4). Although some CpGs were differentially methylated between normal B‐cell populations and CLL samples (Figure S3), no differences in the DNA methylation status of the *RCC1* or *IRF4* locus were detected between CLL with or without the *RCC1::IRF4* translocation. On the other hand, using data from ICGC MMML‐Seq project, expression of *RCC1* was detected in naive B cell and Germinal centre B cells (GCB) cells. We also observed no changes in methylation at the *MED18* locus which was previously reported as epigenetically regulated by SNHG3 in gastric cancer.^10^

*IRF4* is a transcription factor essential for immune cell development. Translocations involving *IRF4* have been described among others in myeloma,^11^ T‐cell lymphoma^12^ and large B‐cell lymphoma.^13^ IRF4 function in CLL is less clear. A locus predisposing to CLL has been mapped to the 3’‐UTR of *IRF4*, with lower expression of IRF4 associated with risk alleles.^14^ Deletion of *IRF4* in Vh11 NZB mice or the B cells of TCL‐1 transgenic mice accelerates the development of spontaneous CLL in these models.^15^, ^16^ Low expression of IRF4 in CLL patients correlates with inferior prognosis.^16^

*RCC1* is a key cell cycle regulator involved in DNA damage response checkpoint with a role in tumourigenesis.^17^ *RCC1* is widely expressed in GCB‐derived B‐cell lymphomas such as follicular lymphoma (FL), diffuse large B‐cell lymphoma (DLBCL) and Burkitt lymphoma (BL) (ICGC MMML‐Seq data, not shown). Remarkably, we detected *RCC1* rearrangement in three other cases from the ICGC MMML‐seq cohort, with partners being *IRF4* in follicular lymphoma and *BCL6* or *ZNF217* in two DLBCL cases respectively (data not shown). Other translocation partners of *RCC1* hitherto published include *PD‐L2/PDCD1LG2/CD273* in primary mediastinal B‐cell lymphoma,^18^ *IGL* and *IGH* in each one CLL case^19^ and *HENMT1* or *ABHD12B* in testicular germ‐cell tumours.^20^ Breaks in all these cases occur in introns 1 or 2 except for the follicular lymphoma case where it occurs in exon 3. Interestingly, the long non‐coding RNA *SNHG3* located at the same locus was previously described as an activation‐induced cytidine deaminase (AID) target.^21^ AID preferentially targets introns 1 and 2 of *SNHG3*,^21^ corresponding to introns 1 and 2 of *RCC1* gene, where most of the breakpoints of the *RCC1::IRF4* translocations are located and where the mutations were found in the cases described herein. Thus, it can be speculated that AID activity might be involved in the generation of the *RCC1::IRF4* translocation.

In summary, we report here 10 additional cases with *t*(1;6)(p35.3;p25.2), including cases with mutated *IGHV*. The translocation results in a fusion transcript between *RCC1* and *IRF4* in the absence of coding mutations. The consequences of this translocation remain unclear. Nevertheless, the detection of translocations in CLL may be important to refine prognostic stratification of patients with CLL.

## AUTHOR CONTRIBUTIONS

S.J. and C.L. contributed equally to the paper with shared first authorship. S.J. and C.L. co‐ordinated the project, performed the research, collected data, analysed data and wrote the paper. N.P., I.N., E.L., S.B., C.D, MSz, MSc and PR performed the research and analysed the data; E.M.M.P., M.J.A. and H.S.W. contributed essential reagents or tools; and L.M., I.W., R.S. and M.J.S.D. initiated the project and designed the research study. All authors reviewed the manuscript.

## FUNDING INFORMATION

This study has been supported by the Bundersministerium fur Bildung und Forschung (BMBF) in the framework of the ICGC MMML‐Seq (01KU1002A‐J), ICGC DE‐Mining (01KU1505G and 01KU1505E), DFG SFB1074 (project B10N), by funds from Cancer Research UK in conjunction with the UK Department of Health on an Experimental Cancer Medicine Centre grant [C10604/A25151], and the Scott‐Waudby Trust (Leicester), Stichting tegen Kanker (Leuven), N. Put and E. Lierman are supported by FWO Vlaanderen. C.L. was supported by an SFB1074 and postdoctoral Beatriu de Pinós grant from Secretaria d'Universitats i Recerca del Departament d'Empresa i Coneixement de la Generalitat de Catalunya and by Marie Sklodowska‐Curie COFUND programme from H2020 (2018‐BP‐00055). The research was carried out at the National Institute for Health and Care Research (NIHR) Leicester Biomedical Research Centre (BRC).

## CONFLICT OF INTEREST STATEMENT

The authors have no relevant conflicts of interest to disclose.

## ETHICS STATEMENT

This study was approved by local Research Ethics Committee and the University Hospitals of Leicester NHS Trust (06/Q2501/122), by the Institutional Review Board of the Medical Faculty of the University of Kiel (A150/10) and University of Ulm (349/11), and University of Leuven (B322201111374).

## PATIENT CONSENT STATEMENT

Samples were obtained after written informed consent.

## Supporting information


Data S1.

## Data Availability

The application to access ICGC‐sequencing data must be completed and submitted through the online submission website. All applicants must review the DACO policies and procedures for details on eligibility, review criteria and office procedures. Additional data that support the findings of this study are available from the corresponding author upon reasonable request.
