# Supplementary material for: The chromosomal translocation t(1;6)(p35.3;p25.2), recurrent in chronic lymphocytic leukaemia, leads to RCC1::IRF4 fusion
Source: Br J Haematol. 2024 Oct 15;205(6):2321–6. doi: 10.1111/bjh.19790 (PMC11637728; doi:10.1111/bjh.19790)
Supplement: Supplementary file 1 — Data S1. [file BJH-205-2321-s001.docx]

**Supplementary Methods**

**Cytogenetic and FISH analysis:** Chromosome analysis was performed by R-banding as described in (1). Karyotypes were described according to the International System for Human Cytogenetic Nomenclature (ISCN) guidelines (2). Interphase fluorescence *in situ* hybridization (FISH) was performed as described in (3). Identification of breakpoints was performed using probes from BACs RP11-290H1 and RP11-442N24 at chromosome 1 and BAC RP11-233K4 at chromosome 6 (Supplementary Figure 2). For the detection of chromosomal abnormalities by FISH affecting IRF4/MUM1 locus, a dual-colour *IRF4/MUM1* break-apart probe was applied using differentially labelled BAC clones, RP3-416J7 and CTD-3232J20 in spectral orange and RP5-1077H22 and RP5-856G1 in spectral green. At the *RCC1* locus, a break-apart probe was applied, using differentially labelled BAC clones, RP11-590G1 and RP11-323G9 in spectrum green, and RP11-318E23 and CTD-3067L23 in spectrum orange. In addition, the *RCC1* probe was labelled in spectrum orange to combine with *IRF4* probe labelled in spectrum green. At least one hundred nuclei were examined whenever possible. The BAC clones used for design of the non-commercial probes were provided by Life Technologies, Darmstadt, Germany. Digital image acquisition, processing, and evaluation were performed using ISIS digital image analysis version 5.0 (MetaSystems, Altussheim, Germany).

**Polymerase Chain Reaction (PCR):** Genomic DNA was extracted using the DNeasy Blood and Tissue kit (Qiagen). *IGHV* sequencing was performed as described(4). *RCC1*::*IRF4* breakpoints PCR reactions were performed using primers designed within exons 1 to 4 of *RCC1* and exons 1 and 2 of *IRF4*. To validate the mutations in *MSH6* and *IL12RB1* identified by WGS, primers were designed to cover the specific mutations. PCR products were subjected to Sanger sequencing using the Big Dye Terminator v1.1 Cycle Sequencing Kit (Life Technologies). Sequence analysis was performed using an ABI PRISM 3130 Genetic Analyzer. Primers sequences and PCR conditions are available upon request.

**Whole genome sequencing:** The experimental and computational procedures for the analysis of WGS data of tumour samples without matched control were performed as previously described(5, 6). The identified changes reported as SNPs in dbSNPs database (http://ncbi.nlm.nih.gov/SNP), which are reported in a public database (gnomAD, 28.09.2018) with allele frequency >0.015, were not considered. Alamut software was used to predict the potential effect of mutations detected applying WGS, using the Sift (http://sift.jcvi.org/, (7)) and Mutation Taster (http://mutationtaster.org/) values.

**DNA methylation:** The Infinium HumanMethylation 450K BeadChip array was performed on three CLL cases with *RCC1*::*IRF4* translocation following manufacturer's instructions. The results were compared with DNA methylation data from Infinium HumanMethylation 450K BeadChip of CLL cases (8) (n=59 cases) and normal B-cells (9-11) (n=92) previously published. Minfi R package (12) was used to analyse and normalise raw idat files of all samples.

**mRNA analysis:** Total RNA was isolated using the RNeasy kit (Qiagen) and residual genomic DNA was digested by DNase I treatment. Superscript II reverse transcriptase (Invitrogen) was used to generate cDNA. Primers sequences and PCR conditions are available upon request.

**Protein expression analysis:** Cells were lysed in lysis buffer (20mM Hepes pH8, 420mM NaCl, 0.5%NP-40, 1mM DTT, protease inhibitors) and protein concentration quantified using Biorad Protein Assay. Antibodies used were anti-RCC1 (ab109379, Abcam), anti-IRF4 (4964S, Cell Signaling Technology), anti-GAPDH (2118S, Cell Signaling Technology)

**Supplementary results**

**
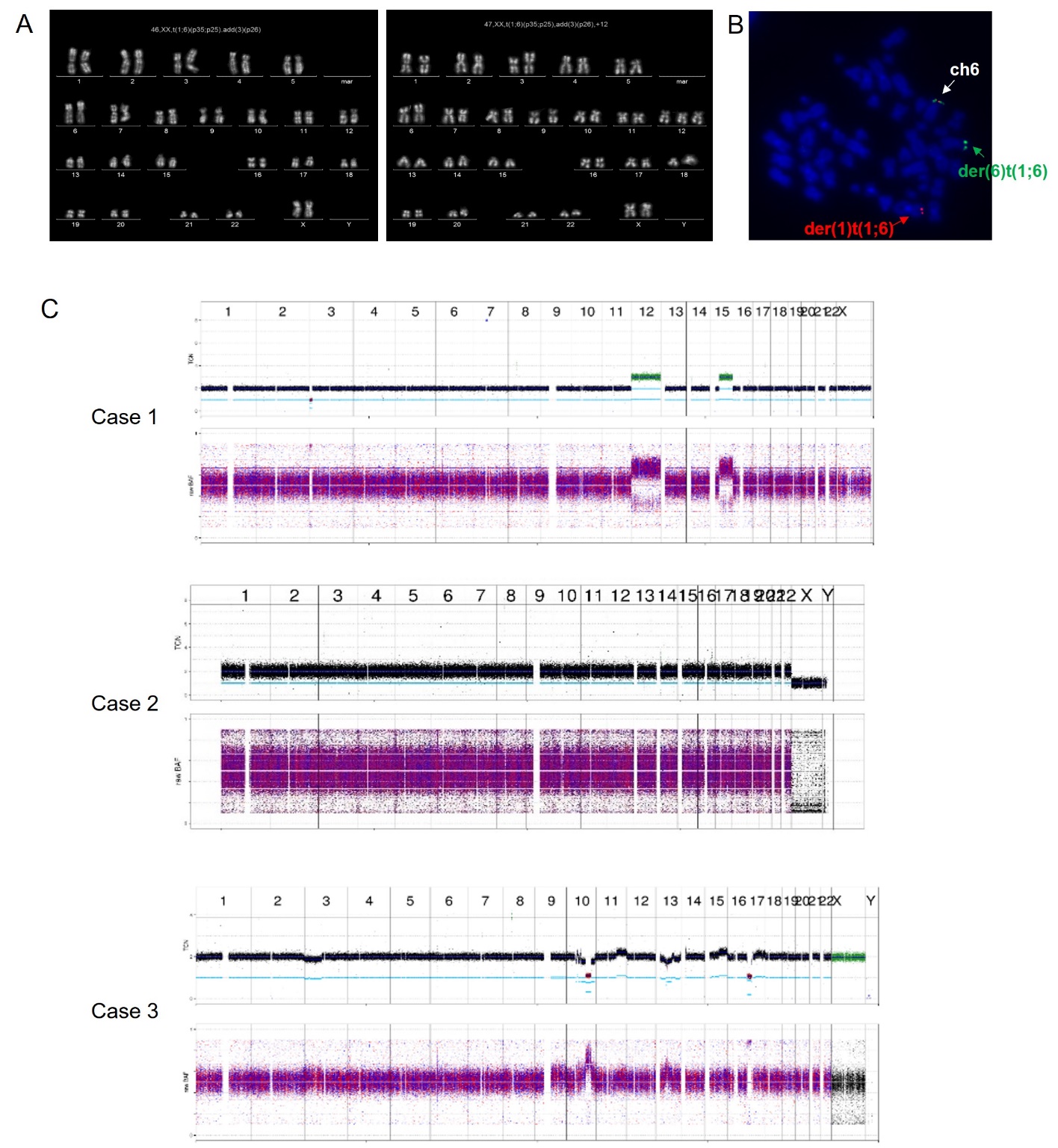
**

**Supplementary Figure 1: Genomic aberrations in *RCC::IRF4* CLL patients**

A) Abnormal diploid female karyotype showing a balanced translocation between the short arms of chromosome 1 and chromosome 6 t(1;6)(p35.3;p25.2), and the addition of unknown material on the short arm of chromosome 3 in all tumour cells and the presence of additional chromosome 12 in 45% of the cells observed in case 1. B) Metaphase FISH analysis of case 1 using dual-colour *IRF4* break-apart probe showed a fusion of the dual-colour probe on the chromosome 6 with an intact *IRF4* locus, an isolated red signal from the telomeric probe on derivative chromosome 1 and a single green signal from the centromeric probe on derivative chromosome 6. C) Imbalance profile of the three *RCC1::IRF4* CLL cases (based on WGS data), losses are indicated in red, gains in green, and copy neutral losses of heterozygosity (LOH) as change in raw BAF. Note gain of chromosomes 12 and 15 in case 1, focal deletion of 13q in case 2 and deletion of 10q and 17p in case 3 among other alterations. Gain of chromosome 15 in case 1 suggests a previously unrecognised der(3)t(3;15)(p25;q11) and loss of 17 confirms a previous dic(14;17)(q10;q10) identified by karyotype.


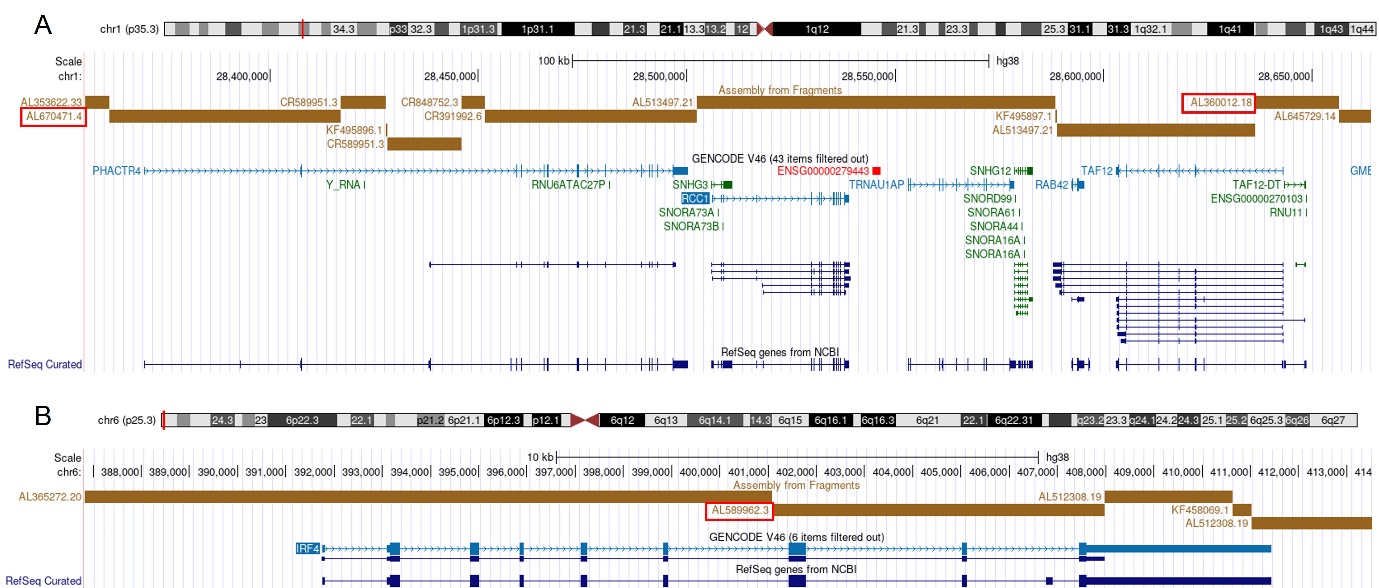


**Supplementary Figure 2: Location of the BACs used to map the translocation t(1;6)(p35.3;p25.2)**

A) Map of chromosome 1 with the region covered by BACs RP11-290H1 (AL670471.4) and RP11-442-N24 (AL360012.18). B) Map of chromosome 6 with the region covered by BAC RP11-233K4 (AL589962.3). The UCSC Genome Browser (http://genome.ucsc.edu) was used to draw this map (13).


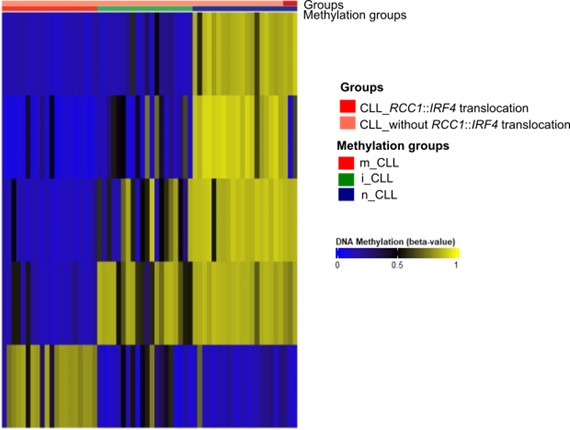


**Supplementary Figure 3: Heat-map of the 5 CpGs used to classify the CLL patients in the three epigenetic groups.**

Methylation value of the 5 CpGs evaluated to classify CLL cases as memory CLL (red), intermediate CLL (green), and naïve CLL (blue) according to previously described epigenetic groups (8) in a panel of 59 CLL cases without the *RCC1::IRF4* translocation (light orange) and the three cases with *RCC1::IRF4* translocation (red).


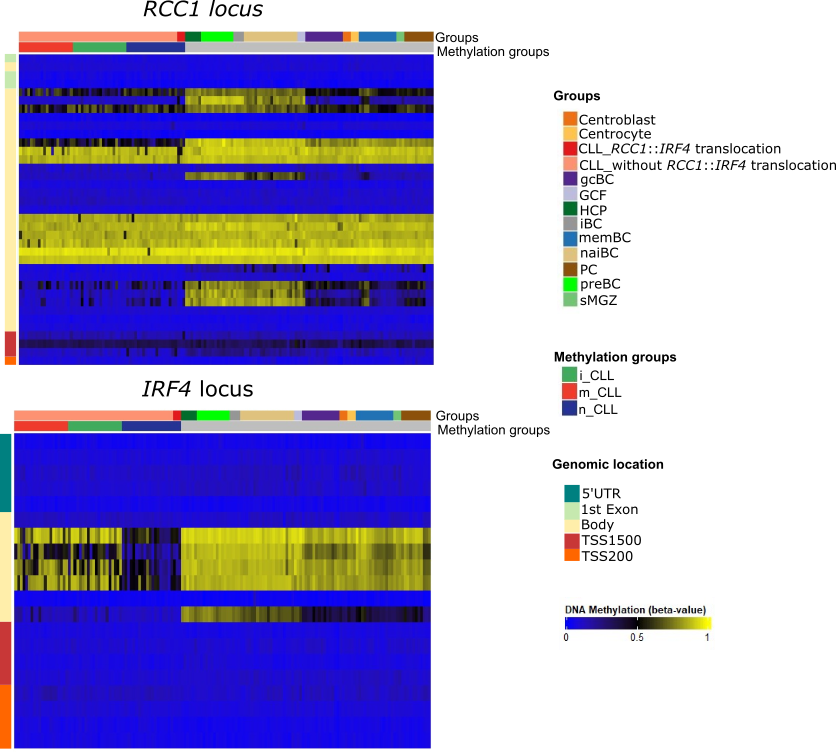


**Supplementary Figure 4: Heat-map showing DNA methylation profiling of *RCC1* and *IRF4* loci in normal B cell and CLL cases with and without *RCC1*::*IRF4* translocation.**

Heat-maps displaying DNA methylation profiling of the *RCC1* and *IRF4* loci. The genomic location of the CpGs analysed for *IRF4* and *RCC1* genes are labelled in different colours based on the genomic annotation, 5’UTR (green), body (white orange), transcription start site (TSS) 200 (orange) and TSS 1500 (red). The normal cells correspond to progenitor cell (HPC), and to immature B cell to mature B cells: immature B cells (iBC), pre B cell (pre-BC), naiBC (naïve B cells), centrocyte, centroblasts, germinal centre founder cells (GCF), germinal centre B cells (gcBC), splenic marginal zone B cells (sMGZ), plasma cells (PC). The CLL cases were classified based on the methylation group and genetic groups. The DNA methylation on *RCC1* locus displays hypomethylation of the 5 CpGs (CpG07807470, CpG15161854, CpG02732067, CpG19409133, and CpG25596287) and hemimethylation of two CpGs (CpG00452755, CpG07807470) analysed in CLL cases compared to precursor B-cells. The DNA methylation on *IRF4* locus shows hypermethylation of the 5 CpGs (CpG07574896, CpG07817739, CpG11417701, CpG17336615), and hypomethylation of the 1 CpG (CpG24319508) analysed in CLL cases compared to precursor B-cells.

Supplementary Table 1. Summary of the main biological characteristics of CLL patients with t(1;6) previously described

| Age | Gender | Stage  (Binet) | WCC  (x10^9^/L) | LDH | *IGHV* | Cytogenetics | Richter | Reference | Used in this study |
| --- | --- | --- | --- | --- | --- | --- | --- | --- | --- |
| 56 | M | B | 17 | - |  | 46,XY,**t(1;6)(p34.1;p23)**[10]/46,XY[10] |  | (14) |  |
| 58 | M | A | 10.7 | N | U | 46,XY,**t(1;6)(p35;p25)**,del(11)(q22q24),+12[2]/46,XY[3] | Y | (15) | L1 |
| 68 | M | C | 70 | High | U | 46,XY,**t(1;6)(p35;p25)**[2]/46,idem,der(17)t(9;17)(q11;p11)[1]/46,idem,der(17;18)(q10;q10)[1] | Y |  | L4 |
| 67 | M | C | 13.6 | N | U | 46,XY,**t(1;6)(p35;p25)**[5]/46,XY[5] |  |  |  |
| 53 | M | B | 12.8 | N | U | 46,XY,**t(1;6)(p35;p25)**,del(11)(q14q23)[4]/46,idem,t(3;17)(q24;q21),del(8)(p21) or inv(8)(p12q12)[4]/46,XY[2] |  |  |  |
| 70 | F | A | 11.8 | N | U | 46,XX,**t(1;6)(p35;p25)**[16]/46,XX[6] |  |  |  |
| 33 | M | B | 34.4 | N | U | 47,XY,**t(1;6)(p35;p25)**,+12[15]/47,XY,der(1)t(1;6)(p35;p25)t(1;2)(?;?),+12[4]/46,XY[7] |  |  |  |
| 57 | M | B | 103 | N | U | 47,XY,**t(1;6)(p35;p25)**,+12[3]/48,idem,+9[5]/47,idem,t(8;9)(q24.3;q11)[5]/47,idem,t(9;15)(q11;p11)[4]/47,idem,t(9;15)(q11;q26)[3]/47,idem,t(9;19)(q11;p13.3[3]/47,idem,t(9;21)(q11;p11)[1]/46,XY[1] | Y |  | L2 |
| 81 | F | B | 10.1 | N | U | 46,X,t(X;19)(q13;q13),**t(1;6)(p35;p25)**,del(4)(q31),add(5)(q35),del(9)(q22q34),-16,add(17)(p13)[7]/46,XX[1] |  |  | L3 |
| 64 | M | - | - | - | - | 44,XY,**t(1;6)(p35;p25)**,der(4)(q21),-9,add(17)(p13),-21,+mar[6]/46,XY[14] |  | (16) |  |
| 75 | F | A | 21.8 | - | - | 47,XX**,t(1;6)(p35;p25)**,+12[13]/46,XX[7] | Y |  |  |
| - | - | - | - | - | - | 45,XY,**t(1;6)(p34.3;p23)**,der(14)?inv(14)(q22q32)t(8;14)(q21.2;q32.3),psu dic(20;17)(p12;p11.2)[2]/45,sl,der(9)t(8;9)(q22;q34)[3]/44,sdl1,-4,der(18)t(4;18)(q12;p11.2)[2]/88,sdl2x2,+9,+9,-der(9)t(8;9)(q22;q34)x2[cp5]/46,XY[8] |  | (17) |  |

Supplementary Table 2. *RCC1* and *IRF4* no coding mutations in *RCC1*::*IRF4* cases analysed using WGS.

| **Case** | **ICGC case** | **Gene** | **Chromosomal location (hg19)** | **Ref.** | **Alt.** | **Variant_type** | **Annovar Function** | **SNPs ID** | **gnomAD Allele Frequency*** |
| --- | --- | --- | --- | --- | --- | --- | --- | --- | --- |
| 1 | 4154480 | *RCC1* | chr1:28834895 | TAGTC | T | indel | intronic | No variants | No variants |
| 2 | 4153126 | *RCC1* | chr1:28842989 | G | C | SNV | intronic | No variants | No variants |
| 3 | 4149243 | *RCC1* | chr1:28834403 | C | G | SNV | intronic | No variants | No variants |
| 3 | 4149243 | *RCC1* | chr1:28834536 | T | TG | indel | intronic | No variants | No variants |
| 2 | 4153126 | *IRF4* | chr6:392209 | C | G | SNV | intronic | No variants | No variants |
| 3 | 4149243 | *IRF4* | chr6:395362 | G | A | SNV | Intronic | rs116544972 | 0.00006493 |
| 3 | 4149243 | *IRF4* | chr6:392531 | C | G | SNV | intronic | No variants | No variants |
| 3 | 4149243 | *IRF4* | chr6:392129 | GCCGTCCAAGGCACCCACAC | G | indel | intronic | No variants | No variants |

*Allele Frequency from European non-Finnish population;

Ref, reference; Alt, alternative

Supplementary Table 3: Recurrent mutations previously described in CLL patients identified in *RCC1::IRF4* CLL cohort.

| **Gene** | **Transcript variant** | **Patient** | **ICGC-case** | **Chromosomal location (hg19)** | **Annovar Function** | **Consequence on protein level** |
| --- | --- | --- | --- | --- | --- | --- |
| *XPO1* | ENST00000404992.2 | 1 | 4154480 | Chr2: 61719186 T>C | Exonic | p.D624G |
| *KLHL4* | ENST00000373119.4 | 1 | 4154480 | chrX: 86887212 A>G | Exonic | p.T443A |
| *NOTCH1** | ENST00000277541.7 | 1 | 4154480 | chr9: 139390648 CAG>C | Exonic | p.P2514Rfs*4 |
| *ATM* | ENST00000278616.4 | 2 | 4153126 | Chr11: 108199892 A>G | Exonic | p.N2412D |
| *MED12L* | ENST00000474524.1 | 3 | 4149243 | Chr3: 151093979 C>T | Exonic | p.Q1309X |
| *TP53* | ENST00000269305.4 | 3 | 4149243 | Chr17: 7578394 T>C | Exonic | p.H179R |

Chr: chromosome; ref: reference; Alt:alternative; Transcipt variants from Ensembl using Genome assembly GRCh38.p13

**NOTCH1* mutation previously detected as WGS analysis by PCR and Sanger sequencing approach used at the diagnosis of the CLL disease.

Supplementary Table 4: Overview of identified coding single nucleotide variants using whole genome sequencing (WGS) analysis in *RCC1-IRF4* cases.

| **Gene** | **Ensembl Transcript** | **Case** | **ICGC-case** | **Chromosomal location (hg19)** | **Annovar Function** | **Consequence on protein level** | **SNPs ID** | **gnomAD Allele Frequency*** | **Alamut** | |
| --- | --- | --- | --- | --- | --- | --- | --- | --- | --- | --- |
|  |  |  |  |  |  |  |  |  | **SIFT (score)** | **Mutation Taster (p-value)** |
| *PMM2* | ENST00000268261.4 | 1 | 4154480 | chr16: 8904998 G>T | Exonic | p.S137I |  |  | Delet. (0.01) | D. Causing (1) |
|  |  | 2 | 4153126 | chr16: 8891804 AG>A | Exonic | p.Q22fs |  |  | n.a. | n.a. |
|  |  | 3 | 4149243 | chr16: 8941582 G>A | Exonic | p.G214D |  |  | Delet. (0) | D. Causing (1) |
| *PRRC2C* | ENST00000338920.4 | 3 | 4149243 | chr1: 171494037 C>T | Exonic | p.S376F | rs527701761 | 0,00004554 | Delet. (0) | D. Causing (0.69) |
|  |  | 2 | 4153126 | chr1: 171553210 G>A | Exonic | p.G2507S | rs150383051 | 0,0001826 | Toler. (0.46) | Polymor. (0.84) |
| *MSH6* | ENST00000540021.1 | 1 | 4154480 | chr2: 48027054 G>C | Exonic | p.R514S | rs34938432 | 0,000189 | Toler. (0.75) | Polymor. (1) |
|  |  | 3 | 4149243 | chr2: 48010431 C>T | Exonic | p.A20V | rs63750664 | 0 | Toler. (0.28) | Polymor. (1) |
| *ANKRD36* | ENST00000420699.2 | 2 | 4153126 | chr2:97858609 C>A | Exonic | p.P788T |  |  | Toler. (0.66) | n.a. |
|  |  | 3 | 4149243 | chr2: 97877405 G>A | Splicing |  | rs769522252 | 0,00001 |  |  |
| *WFS1* | ENST00000226760.1 | 2 | 4153126 | chr4: 6303119 C>T | Exonic | p.P533S | rs146132083 | 0,001398 | Delet. (0) | D.Causing (1) |
|  |  | 3 | 4149243 | chr4: 6292941 G>A | Exonic | p.E160K |  |  | Toler. (0.25) | Polymor. (0.705) |
| *PCDHGA6* | ENST00000517434.1 | 2 | 4153126 | chr5: 140753782 C>A | Exonic | p.F44L | rs770866531 | 0,00003581 | n.a. | Polymor. |
|  |  | 3 | 4149243 | chr5: 140753843 C>G | Exonic | p.R65G |  |  |  | Polymor. |
| *PCDHGA7* | ENST00000518325.1 | 1 | 4154480 | chr5: 140764616 G>A | Exonic | p.R717Q | rs762319044 | 0 | Toler. (0.29) | Polymor. (1) |
|  |  | 2 | 4153126 | chr5: 140762945 C>A | Exonic | p.P160Q |  |  | Toler. (0.15) | Polymor. (0.99) |
| *VWA5A* | ENST00000456829.2 | 1 | 4154480 | chr11: 123993769 G>A | Exonic | p.R288H | rs763589021 | 0,00001579 | Toler. (0.1) | D. Causing (0.99) |
|  |  | 3 | 4149243 | chr11: 124015993 A>T | Exonic | p.H735L |  |  | Delet. (0.01) | D. Causing (0.96) |
| *FANCM* | ENST00000542564.2 | 2 | 4153126 | chr14: 45606310 A>C | Exonic | p.S183R | rs368937236 | 0,0009336 | Delet. (0.01) | Polymor. (1) |
|  |  | 3 | 4149243 | chr14: 45665640 C>A | Exonic | p.S1843Y |  |  | Delet. (0) | D. Causing (0.97) |
| *STARD9* | ENST00000290607.7 | 2 | 4153126 | chr15: 42985431 G>T | Exonic | p.K3885N |  |  | Toler. (0.09) | Polymor. (1) |
|  |  | 3 | 4149243 | chr15: 42957491 T>C | Exonic | p.I406T | rs577186934 | 0,0001904 | Delet. (0) | D. Causing (0.99) |
| *OSGIN1* | ENST00000361711.3 | 2 | 4153126 | chr16: 83994622 C>T | Exonic | p.Q145X |  |  | n.a. | n.a. |
|  |  |  |  | chr16: 83999596 C>T | Exonic | p.T473I |  |  | Toler. (0.18) | Polym. (1) |
|  |  | 3 | 4149243 | chr16: 83998779 G>A | Exonic | p.V201M | rs138968808 | 0.00006337 | Delet. (0.01) | D. Causing (1) |
| *ZNF469* | ENST00000437464.1 | 1 | 4154480 | chr16: 88501357 G>C | Exonic | p.R2465S |  |  | Toler. (0.22) | Polymor. (0.99) |
|  |  | 2 | 4153126 | chr16: 88505303 G>A | Exonic | p.E3781K | rs201834513 | 0.001378 | Delet. (0.05) | Polymor. (0.76) |
| *SCN4A* | ENST00000578147.1 | 1 | 4154480 | chr17: 62026831 T>C | Exonic | p.K971E |  |  | Delet. (0.01) | Polymor. (0.89) |
|  |  | 2 | 4153126 | chr17: 62028933 C>T | Exonic | p.G902S | rs200517944 | 0,0003316 | Toler. (0.13) | Polymor. (1) |
| *IL12RB1* | ENST00000600835.2 | 1 | 4154480 | chr19: 18191732 C>G | Exonic | p.V107L |  |  | Delet. (0.05) | n.a. |
|  |  | 3 | 4149243 | chr19: 18177411 G>A | Exonic | p.P475L |  |  | Delet. (0) | n.a. |
| *VN1R2* | ENST00000341702.3 | 1 | 4154480 | chr19: 53762139 G>A | Exonic | p.V171I |  |  | n.a. | Polymor. |
|  |  | 2 | 4153126 | chr19: 53762097 G>T | Exonic | p.D157Y | rs61751885 | 0.0009714 | n.a. | Polymor. |

*Allele Frequency from European non-Finnish population. Delet.: deleterious; Polymor.: polymorphism; D.: disease; Toler.: tolerated; n.a.: not available

Supplementary Table 5 Overview of *MSH6* and *IL12RB1* mutations and the respective PCR primers and PCR conditions for Sanger sequencing validations.

| **Gene** | **Patient** | **ICGC-case** | **Chromosomal location (hg19)** | **Consequence on protein level** | **Annovar Function** | **Forward primer (5’-3’)** | **Annealing Temp.^1^** | **PCR^2^(bp)** |
| --- | --- | --- | --- | --- | --- | --- | --- | --- |
|  |  |  |  |  |  | **Reverse primer (5’-3’)** |  |  |
| *MSH6* | 1 | 4154480 | Chr2: 48027054 G>C | p.R514S | exonic | TCATTGTCCTGTTCTCTTCAGG | 60°C | 214 |
|  |  |  |  |  |  | CAGAGAGGGCCAATTCACTT |  |  |
|  | 3 | 4149243 | Chr2: 48010431 C>T | p.A20V | exonic | AGATGCGGTGCTTTTAGGAG | 60°C | 285 |
|  |  |  |  |  |  | CCCTCCGTTGAGGTTCTTC |  |  |
| *IL12RB1* | 1 | 4154480 | Chr19: 18191732 C>G | p.V107L | exonic | CTGATGGCCTCTCTGGGTAA | 60°C | 247 |
|  |  |  |  |  |  | CACTGACACCCTCCTTCCTG |  |  |
|  | 3 | 4149243 | Chr19: 18177411 G>A | p.P475L | exonic | CCTGACACCTGTTTGCTGTC | 60°C | 192 |
|  |  |  |  |  |  | TGGTGTTGACTATGACAATGGT |  |  |

Gene *MSH6*: ENST00000540021.1 and *IL12RB1*: ENST00000600835.2. ^1^ Annealing temperature in °C, ^2^ Length of PCR product in base pairs (bp)

Supplementary references

1. Schlegelberger B, Metzke S, Harder S, Zühlke-Jenisch R, Zhang Y, Siebert R. Classical and Molecular Cytogenetics of Tumor Cells. In: Wegner R-D, editor. Diagnostic Cytogenetics. Berlin, Heidelberg: Springer Berlin Heidelberg; 1999. p. 151-85.

2. Shaffer LGM-J, J.; Schmid, M. ISCN 2013 : an international system for human cytogenetic nomenclature (2013). Switzerland: Basel : Karger, c2013; 2013.

3. Martin-Subero JI, Harder L, Gesk S, Schlegelberger B, Grote W, Martinez-Climent JA, et al. Interphase FISH assays for the detection of translocations with breakpoints in immunoglobulin light chain loci. Int J Cancer. 2002;98(3):470-4.

4. van Dongen JJ, Langerak AW, Bruggemann M, Evans PA, Hummel M, Lavender FL, et al. Design and standardization of PCR primers and protocols for detection of clonal immunoglobulin and T-cell receptor gene recombinations in suspect lymphoproliferations: report of the BIOMED-2 Concerted Action BMH4-CT98-3936. Leukemia. 2003;17(12):2257-317.

5. Hubschmann D, Jopp-Saile L, Andresen C, Kramer S, Gu Z, Heilig CE, et al. Analysis of mutational signatures with yet another package for signature analysis. Genes Chromosomes Cancer. 2021;60(5):314-31.

6. Lopez C, Kleinheinz K, Aukema SM, Rohde M, Bernhart SH, Hubschmann D, et al. Genomic and transcriptomic changes complement each other in the pathogenesis of sporadic Burkitt lymphoma. Nat Commun. 2019;10(1):1459.

7. Kumar P, Henikoff S, Ng PC. Predicting the effects of coding non-synonymous variants on protein function using the SIFT algorithm. Nat Protoc. 2009;4(7):1073-81.

8. Kulis M, Heath S, Bibikova M, Queiros AC, Navarro A, Clot G, et al. Epigenomic analysis detects widespread gene-body DNA hypomethylation in chronic lymphocytic leukemia. Nat Genet. 2012;44(11):1236-42.

9. Oakes CC, Seifert M, Assenov Y, Gu L, Przekopowitz M, Ruppert AS, et al. DNA methylation dynamics during B cell maturation underlie a continuum of disease phenotypes in chronic lymphocytic leukemia. Nat Genet. 2016;48(3):253-64.

10. Lee ST, Xiao Y, Muench MO, Xiao J, Fomin ME, Wiencke JK, et al. A global DNA methylation and gene expression analysis of early human B-cell development reveals a demethylation signature and transcription factor network. Nucleic Acids Res. 2012;40(22):11339-51.

11. Kulis M, Merkel A, Heath S, Queiros AC, Schuyler RP, Castellano G, et al. Whole-genome fingerprint of the DNA methylome during human B cell differentiation. Nat Genet. 2015;47(7):746-56.

12. Aryee MJ, Jaffe AE, Corrada-Bravo H, Ladd-Acosta C, Feinberg AP, Hansen KD, et al. Minfi: a flexible and comprehensive Bioconductor package for the analysis of Infinium DNA methylation microarrays. Bioinformatics. 2014;30(10):1363-9.

13. Nassar LR, Barber GP, Benet-Pages A, Casper J, Clawson H, Diekhans M, et al. The UCSC Genome Browser database: 2023 update. Nucleic Acids Res. 2023;51(D1):D1188-D95.

14. Harada K, Ikeda K, Matsumoto H, Furukawa M, Takahashi H, Ohkawara H, et al. A Japanese case of chronic lymphocytic leukemia with t (1;6). Exp Hematol Oncol. 2012;1(1):28.

15. Michaux L, Wlodarska I, Rack K, Stul M, Criel A, Maerevoet M, et al. Translocation t(1;6)(p35.3;p25.2): a new recurrent aberration in "unmutated" B-CLL. Leukemia. 2005;19(1):77-82.

16. Rodrigues Pereira Velloso E.D., Borri D., Alonso Ratis C., Fleury Perin G., Hamerschlak N., Bacal N.S., et al. Chronic lymphocytic leukaemia/Small lymphocytic lymphoma (CLL/SLL) associated with translocation t(1;6)(p35;p25) as part of complex karyotype. Atlas Genet Cytogenet Oncol Haematol. 2011;15:467–9.

17. Schultz RA, Delioukina M, Gaal K, Bedell V, Smith DD, Forman SJ, et al. Evaluation of chronic lymphocytic leukemia by BAC-based microarray analysis. Mol Cytogenet. 2011;4(1):4.
